# Pathogenicity of Duck Adenovirus Type 3 in Chickens

**DOI:** 10.3390/ani14162284

**Published:** 2024-08-06

**Authors:** Xiwen Zhang, Bin Xu, Huiqin Zhou, Xiang Zhou, Qingfeng Wang, Jiayu Sun, Kewei Liu, Lisha Zha, Jinchun Li, Yin Dai, Fangfang Chen

**Affiliations:** 1 Key Laboratory of Veterinary Pathobiology and Disease Control, College of Animal Science and Technology, Anhui Agricultural University, Hefei 230036, China; ww05140317ww@163.com (X.Z.); 15656598670@163.com (B.X.); zhouhuiqin67@163.com (H.Z.); zx201411864@outlook.com (X.Z.); wangqingfeng2022@163.com (Q.W.); sjyqwer0113@163.com (J.S.); l2570859842@163.com (K.L.); zhalisha@ahau.edu.cn (L.Z.); jinchunli64@163.com (J.L.); 2 Institute of Animal Husbandry and Veterinary Science, Anhui Academy of Agricultural Sciences, Hefei 230036, China; daiyin2020@163.com

**Keywords:** duck adenovirus Type 3 (DAdV-3), chicken, pathogenicity, mutation

## Abstract

**Simple Summary:**

This study aimed to evaluate the pathogenicity of duck adenovirus Type 3 (DAdV-3) in chickens. In this study, this virus was identified through the amplification of specific viral fragments and whole-genome sequencing, and its pathogenicity was identified by means of its inoculation into chickens. The isolated DAdV-3 led to higher pathogenicity and fatality rates in chickens. The main clinical symptoms in the sacrificed chicks were hepatitis–hydropericardium syndrome (HHS), swollen and petechial hemorrhages of the bursa, and the gizzard’s endothelium falling off easily. Histopathological examination showed swelling, necrosis, lymphocyte infiltration, and basophilic inclusion bodies in multiple organs. Higher antibody levels were found in the surviving chickens. The major variation domains (ORF19B, ORF66, and ORF67) did not change after infection with the virus in Muscovy ducks and chickens. This is the first study to report the pathogenicity of DAdV-3 in chickens, and it provides an experimental basis for newly infected hosts of DAdV-3.

**Abstract:**

Duck adenovirus Type 3 (DAdV-3) severely affects the health of ducks; however, its pathogenicity in chickens remains unknown. The objectives of this study were to evaluate the pathogenicity and major pathological changes caused by DAdV-3 in chickens. Viral DNA was extracted from the liver of the Muscovy duck, and the *fiber-2* and *hexon* fragments of DAdV-3 were amplified through polymerase chain reaction (PCR). The evolutionary tree revealed that the isolated virus belonged to DAdV-3, and it was named HE-AN-2022. The mortality rate of chicks that received inoculation with DAdV-3 subcutaneously via the neck was 100%, while the mortality rate for eye–nose drop inoculation was correlated with the numbers of infection, with 26.7% of chicks dying as a result of exposure to multiple infections. The main symptoms exhibited prior to death were hepatitis–hydropericardium syndrome (HHS), ulceration of the glandular stomach, and a swollen bursa with petechial hemorrhages. A histopathological examination revealed swelling, necrosis, lymphocyte infiltration, and basophilic inclusion bodies in multiple organs. Meanwhile, the results of quantitative real-time PCR (qPCR) demonstrated that DAdV-3 could affect most of the organs in chickens, with the gizzard, glandular stomach, bursa, spleen, and liver being the most susceptible to infection. The surviving chicks had extremely high antibody levels. After the chickens were infected with DAdV-3 derived from Muscovy ducks, no amino acid mutation was observed in the major mutation regions of the virus, which were ORF19B, ORF66, and ORF67. On the basis of our findings, we concluded that DAdV-3 infection is possible in chickens, and that it causes classic HHS with ulceration of the glandular stomach and a swollen bursa with petechial hemorrhages, leading to high mortality in chickens. The major variation domains did not change in Muscovy ducks or in chickens after infection. This is the first study to report DAdV-3 in chickens, providing a new basis for preventing and controlling this virus.

## 1. Introduction

Aviadenoviruses are nonenveloped viruses that contain double-stranded DNA molecules that are 33–45 kbp in size, including at least 14 species [1]. Birds are the unique hosts for aviadenoviruses [2], and FAdV-A (FAdV-1), FAdV-C (FAdV-4), FAdV-E (FAdV-8a and 8b), DAdV-A(DAdV-1), DAdV-B (DAdV-2 and 3), and DAdV-4 can infect waterfowl [3,4]. According to the taxonomy released by the International Committee on Taxonomy of Viruses (ICTV), published in 2022, there are two species of duck adenovirus: A (DAdV-A) and B (DAdV-B) (ictv. global/report/adenoviridae). The former, also known as duck Adenovirus A (DAdV-1), is responsible for the egg-drop syndrome virus (EDSV) in laying hens [5]. The latter includes DAdV-2 and 3, which belong to the same branch as goose Adenovirus 4 (GoAdV-4) [6,7]. DAdV-4, which was first isolated in China in 2020, is a separate type that falls somewhere in the middle of the other kinds of avian adenoviruses (FAdVs) and DAdV-B [3]. The whole-genome sequence identity of the isolated DAdV-3 with DAdV-2 and -4 was 92.3% and 57.2%, respectively. Although these adenoviruses were isolated from ducks, their pathogenicity varies greatly, with DAdV-3 exhibiting the highest incidence and mortality. DAdV-3 (GD-CH-2014) has 99–100% genomic sequence identity and a fatality rate of 35–43%. Infected ducks exhibit yellowing and hemorrhaging in the liver, along with slight pericardial effusion, swelling, and hemorrhaging in the kidneys. DAdV-3 has been found in several Chinese provinces, such as Guangdong, Fujian, Zhejiang, and Anhui, and has been responsible for significant economic losses within the duck farming industry since it was first reported in 2014 [7,8,9]. In contrast, DAdV-4 has not yet caused serious disease outbreaks among ducks [3].

DAdV-3 is a nonenveloped, double-stranded DNA virus. The capsid consists of hexon and penton, and the latter consist of the penton base and two fibers (fiber 1 and fiber 2) [1,7]. Similar to FAdV-4, DAdV-3 has two fiber proteins: a short fiber 1 and a longer fiber 2. The former can directly mediate the infection of DAdV-3, while the latter can induce neutralizing antibodies and is an effective protective immunogen that offers complete protection against DAdV-3 infection [8,10,11]. Hexon, the principal ingredient of the capsid, is the basis for the classification of DAdVs. Penton creates a bond between the fiber and the hexon protein, but currently, its role in DAdV-3 is unclear.

It is unclear whether the virus can infect chickens and, if so, whether it will present differently than it does in waterfowl. In this study, we investigated the characteristics of virus-infected chickens, laying a foundation for the transmission characteristics of DAdV-3 among different birds, the prevention and control of this virus, and research into and application of vaccines.

## 2. Materials and Methods

### 2.1. Animals and Ethical Statement

One-day-old specific-pathogen-free (SPF) chickens, SPF chicken embryos, and SPF duck embryos were purchased from Lihua Agricultural Co., Ltd. (Lihua, Zhejiang, China). The SPF chicken embryos’ incubation temperatures were as follows: 31–36 °C for 1-week-old embryos; 2 weeks, 29–31 °C for 2-week-old embryos; 3 weeks, 27–29 °C for 3-week-old embryos; and 22–27 °C for embryos aged 4 weeks or older. The humidity was 40%. In addition, an 8-week-old SPF rabbit was purchased from Anhui Medical University (Hefei, Anhui, China). The animal use protocol described below was reviewed and approved by the Institutional Animal Care and Use Committee (IACUC), Anhui Agriculture University (AHAUB202404).

### 2.2. Viruses

The DAdV-3 in this study, A/Muscovy duck/Anhui/DK2022 (DK2022), was isolated from the liver tissue of Muscovy ducks (*Cairina moschata*) at a duck farm in Anhui in 2022. Total DNA and RNA were extracted from the liver of the Muscovy duck using a viral RNA/DNA purification kit (Tiangen, Beijing, China) according to the manufacturer’s instructions. For the *hexon* and *fiber-2* genes of DAdV-3, two pairs of primers were designed to amplify the target genes; the sequence and fragment sizes of the primers are shown in Table 1. In addition to detection of DAdV-3, the sample was also tested for other potential pathogens, such as FAdV-4 (stored by our lab), duck astrovirus (DAstV), duck Tembusu virus (DTMUV), avian pulmonary virus (aMPV), avian influenza virus (AIV), and duck circovirus (DuCV), as described in other studies [12,13,14,15,16]. Meanwhile, we designed *fiber* gene primers of DAdV-1, -2, and -4 for PCR amplification; the primer sequences are shown in Table 1. The liver tissue samples were homogenized in sterile phosphate-buffered saline (PBS) (pH 7.2), resulting in a 20% suspension (w/v); were freeze–thawed three times; and then were centrifuged at 10,000× *g* for 10 min. The supernatant was filtered through a 0.22 µm filter and diluted 10 times with a cold PBS gradient. The virus was isolated via the inoculation of 8-day-old SPF chicken embryos and 10-day-old SPF duck embryos. The filtered tissue supernatants were subsequently passaged three times with the inoculation allantoic cavity of 8-day-old SPF chicken embryos (0.1 mL/embryo) or the allantoic cavity of 10-day-old SPF duck embryos (0.2 mL/embryo) through a limiting dilution assay [17]. The aseptically collected allantoic fluid from dead embryos was the third-generation virus. The preparation of allantoic fluid from SPF chick embryos or duck embryos was performed as previously described [18]. The chicken hepatocellular carcinoma (LMH) cells were cultured in a culture medium DMEM/F12 (Biosharp, Beijing, China) containing 10% FBS (GE, Salt Lake City, UT, USA). After overnight incubation, the cell monolayers were washed twice with sterile PBS and inoculated with the filtered tissue supernatant for 90 min at 37 °C. After adsorption, DMEM/F12 supplemented with 2% FBS was added. The cultures were incubated at 37 °C with 5% CO_2_ and passaged every 3 days. The third-generation virus was used to calculate the virulence. We also used the same method to determine the virulence of viruses from chicken and duck embryos. The 50% infectious dose (TCID50) of the tissue culture of the virus was determined through infection of the LMH, and the values were calculated using the Reed–Muench method [19].

### 2.3. Genetic and Phylogenetic Analyses

One gram of Muscovy duck liver was taken and the genomic DNA of DAdV-3 was extracted using a DNA extraction kit (Anheal, Beijing, China). The quality of the genomic DNA was measured using a TBS-380 fluorometer (Turner BioSystems Inc., Sunnyvale, CA, USA). When the optical density (OD) 260/280 ratio was 1.8 to 2.0, 6 µg of DNA was sent to Biozeron Biotechnology (Shanghai, China) for gene sequencing and genome assembly. The assembled genome sequence was submitted to NCBI(OP432083). The BLAST algorithm search tool (BLASTn) and basic local alignment (BLASTp) were used to compare the nucleotide and protein sequences of the genome, respectively, against the NCBI GenBank database. The obtained amino acid sequences were compared with the reported DAdV-2 (KJ469653) and mapped. The hexon amino acid sequence of DAdV-3 was compared with the hexon amino acid sequence of 18 strains of avian adenovirus strains, FAdV-A, -B, -C, -D, -E, DAdV-A, and DAdV-B in NCBI for phylogenetic analysis. Phylogenetic trees were generated using the neighbor-joining (NJ) method in MEGA 11 version software (https://www.megasoftware.net/).

### 2.4. Gene Amplification and Amino Acid Comparison Analysis of Major Differential Domains of DAdV-3

The major variation domains of the isolated DAdV-3 (HF-AH-2022) and the nonvirulent strain DAdV-3 (HF-AH-2020) were compared and amplified. In previous studies, a nonvirulent strain DAdV-3 called HF-AH-2020 was isolated [9], and MEGA 11 was applied to compare the genome of two DAdV-3 strains labeled HF-AH-2020 and HF-AH-2022. The main changes occurred in regions between the different the virulent DAdV-3 strains ORF19B and ORF66. Some scholars found that ORF67 exhibited the most notable difference between different strains of DAdV-3 [20]. In this study, PCR was used to amplify the three ORF regions, ORF19B, ORF66, and ORF67, from viruses isolated from chickens and Muscovy ducks infected with HF-AH-2022, and MEGA 11 was used to compare the differences. Primers were designed for the amplification and comparison of these regions, and their sequences are shown in Table 1.

**Table 1:** The sequences of the primers used in this study.

Amplified Gene	Primer Sequences (5’–3’)	Size, bp
*fiber-2 (F)*	F: acgtcaccgatcccatcatc R: attgacggtcgtggcattgt	683 bp
*h* *exon*	F: atggccgctctgacccctga R: attcagccttagctactttc	640
*f* *iber* *-* *2*	F: gcttcgcgactatttcaacca R: gccttaacgactgcggtttc	127
*ORF 66*	F: atgggaatgtagatcgtggtg R: ttttgctgggatcctcaacct	420
*ORF 67*	F: acctaagccacccctaccag R: gcaggatacgtcaccacgat	398
*ORF 19B*	F: tggtggtggaaattgatgaaga R: ttggcagtcagtgtgattcct	307
*fiber (DAdV-1)*	F: ctgacccaagatggtgaat R: gcaacctttgctgctttgttc	403
*fiber (DAdV-2)*	F: ggtcttggagtagtagtaaac R: ccaacccgtcattatttcttatc	418
*fiber-2 (DAdV-4)*	F: tccacctagacgaaactatg R: gtcgtggcgtcgttgtcgc	532

Note: The restriction site is underlined. F, forward primer; R, reverse primer.

### 2.5. Pathogenicity of the HF-AN-2022 Strain in Chickens

In our experiment, One hundred and twenty 15- and 30-day-old SPF chickens were randomly divided into 14 groups (n = 15 per inoculated group). Two inoculation routes were used: subcutaneous injection via the neck and administration via eye–nose drops. The third-generation virus from chicken embryos was inoculated, and the method, dosage, and frequency of inoculation are shown in Table 2. Groups 1–4 were subcutaneously inoculated; chicks in Groups 1 and 2 were inoculated with 0.2 mL of chick embryo allantoic fluid containing 2 × 10^5.41^ TCID50/0.1 mL, whereas chicks in Groups 3 and 4 were inoculated with 0.2 mL of PBS (Beyotime, Shanghai, China) in the same manner as the negative controls. Groups 5–10 were inoculated with eye–nose drops; of these, Groups 5 and 7 were inoculated with 0.2 mL only once, whereas Groups 6 and 8 were inoculated with 0.2 mL each time twice daily for three consecutive days, and chicks in Groups 9 and 10 were inoculated with the same dose of PBS in the same manner as the negative controls.

Meanwhile, chicks in Groups 11 and 12 were inoculated with allantoic fluid from duck embryos (third-generation virus). The methods, dosage, and frequency of inoculation are shown in Table 2. Chicks in Groups 13 and 14 were inoculated with the same dose of PBS in the same manner as the negative controls. All chickens were observed for 12 d. At 3–5 days post-inoculation (dpi), the heart, liver, spleen, lung, kidney, gizzard, glandular stomach, brain, duodenum, and bursa of three of the sacrificed chickens in Group 2 were collected separately. In Group 4, three chickens were randomly selected, and the same tissues were extracted after euthanasia to be used as the negative control. Sedation was performed via anesthesia using a Rompun–ketamine (1 mg/kg) mixture administered via intramuscular injection into the thigh, followed by an intravenous injection of pentobarbital (30 mg/kg; Sigma-Aldrich, St. Louis, MO, USA) into the wing. All chickens were kept according to standard protocols, and data, such as clinical signs and symptoms, were collected daily. Organs from these chickens were collected for histopathological assays and viral DNA load assays.

### 2.6. Histopathological Assays

Tissue samples were obtained from the heart, liver, spleen, lung, kidney, gizzard, glandular stomach, muscle, brain, duodenum, and bursa of the sacrificed birds, fixed in a 4% paraformaldehyde fixing solution at pH 7.4, and then embedded in paraffin blocks. Furthermore, these tissues were sliced into 3 µm thick sections using a Microm HM 360 microtome (Microm Laborgeräte GmbH, Walldorf, Germany). A 3 µm thick tissue section was prepared by using a Mi-crom HM 360 microsectioning mechanism. Sections were mounted on glass slides and stained with hematoxylin and eosin. Pictures were taken using a bright-field microscope, and photomicrographs were taken with a DP25 digital camera (Olympus, Tokyo, Japan).

### 2.7. Viral DNA Load in the Infected Samples

To analyze the distribution of DAdV-3 in infected chickens, after 3–5 dpi, tissues were collected from three sacrificed chickens from Group 2. Samples were taken from the heart, liver, spleen, kidney, brain, gizzard, glandular stomach, duodenum, lung, and bursa; they were then frozen in liquid nitrogen and stored at −80 °C. Fifty milligrams was taken from each tissue for DNA extraction, which was implemented according to a previously described method [9]. The viral load in different tissues was detected through quantitative real-time PCR (qPCR). SYBR qPCR with primers annealing within the highly conserved *fiber-2* region was performed to determine the viral load, as shown in Table 1. The virus was quantified through qPCR using the AceQ^®^ qPCR SYBR^®^ Green Master Mix (Vazyme, Nanjing, China). Standard curves were obtained by using 10-fold serial dilutions of a linearized plasmid containing the partial *fiber-2* gene of DAdV-3 and were run three times in duplicate. Negative and no-template controls were included during preparation of the samples and qPCR to monitor possible contamination. The number of viral genome copies per reaction was calculated by comparing the threshold cycle (CT) values of the investigated samples’ threshold CT values with the standard curves. An assessment of the specificity of the qPCR products was accomplished by analyzing the melting curve. Each reaction was performed in triplicate, and the results are expressed as the mean  ±  standard deviation (SD). The SD value of each organ was used as the final copy number. Finally, the viral load was calculated as the number of viral copies per gram of tissue.

### 2.8. ELISA

Before the experiment, the indirect enzyme-linked immunosorbent assay (ELISA) method was established to determine the levels of DAdV-3-specific antibodies in the serum of surviving chickens. Twenty milliliters of positive allantoic solution from chicken embryos was collected to purify the virus by ultracentrifugation, and centrifugation at 134,000× *g* for 4.5 h was performed to obtain 1 mL of concentrated allantoic solution. The protein concentration of the virus was detected with the BCA protein concentration assay kit (Beyotime, Shanghai, China). Rabbits were immunized by multiple subcutaneous injections (600 µg/kg body weight) on Days 0, 14, and 28 after the solution was emulsified and mixed with the adjuvant (white oil/glycerin = 3:1). On the 14th day after the third immunization, blood was collected, serum was separated through the auricular vein of the rabbit, and serum was obtained as a positive control. Meanwhile, the rabbits in the blank group were injected with PBS only, and serum was collected in the same manner at the same time for the negative control. The optimal dilution of the antigen and serum was determined by a checkerboard titration with rabbit DAdV-3-positive and -negative sera. The obtained virus, diluted in a 0.05 M carbonate buffer (pH 9.6), was coated separately on ELISA plates (Shenggong, Shanghai, China) in amounts ranging from 0.125 to 4 µg/µL. The dilutions of rabbit serum ranged from 1:500 to 1:120,000. Both the reference positive and negative sera were serially diluted twofold and tested on separate plates. The dilutions that resulted in the maximum difference in absorbance at 450 nm for the positive and negative sera (P/N) were defined as the optimal working conditions to test the experimental samples of chicken serum (Groups 8 and 10). After blood samples were collected, the living animals were anesthetized via an intravenous injection of sodium pentobarbital.

The level of DAdV-3 antibodies in chicken serum was detected using ELISA. The specific detection method was performed as previously described [9]. The cutoff value was an OD of 0.2 at 450 nm. In order to ensure the accuracy of the measurement of antibody titers, the OD values of the samples (ODsample) and the positive control (ODpositive) needed to be subtracted from the OD values of the negative control (ODnegtive) for correction. The sample’s value was calculated according to the formula: value = (ODsample − ODnegtive)/(ODpositive − ODnegtive). Each sample of chicken serum was analyzed in triplicate. After 30 days of inoculation, chickens were euthanized by means of CO_2_ inhalation when they reached a predetermined humane endpoint.

## 3. Results

### 3.1. Identification and Phylogenetic Analysis of DAdV-3

**Figure 1:**
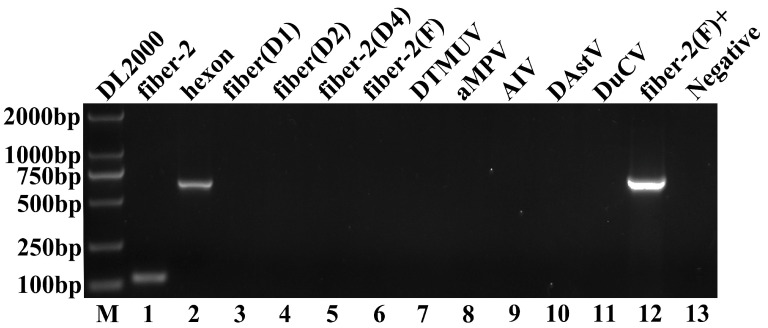
The *fiber-2* and *hexon* gene fragments of DAdV-3 were amplified via PCR. Lanes 1–11 were the product of DAdV-3-fiber-2, DAdV-3-hexon, DAdV-1, DAdV 2, DAdV 4, FAdV-4, DTMUV, aMPV, AIV, DAstV, and DuCV. Lane 12 was the FAdV-4-positive sample, and Lane 13 was the negative control.

A suspected DAdV-3 sample was isolated from the liver of a Muscovy duck. The results showed that specific bands at 640 and 127 bp were obtained by means of PCR amplification. The obtained *hexon* and *fiber-2* gene sequences shared 100% sequence identity with DAdV-3 (HF-AN-2020), referred to as HF-AH-2022. In contrast, the sample tested negative for FAdV-4, DTMUV, aMPV, AIV, DAstV, DuCV, DAdV-1, DAdV-2, and DAdV-4, with the exception of the FAdV-4-positive sample (683 bp), as shown in Figure 1.

### 3.2. Whole-Genome Sequence of the Virus and Detection of DAdV-3 Mutant Amino Acids

**Figure 2:**
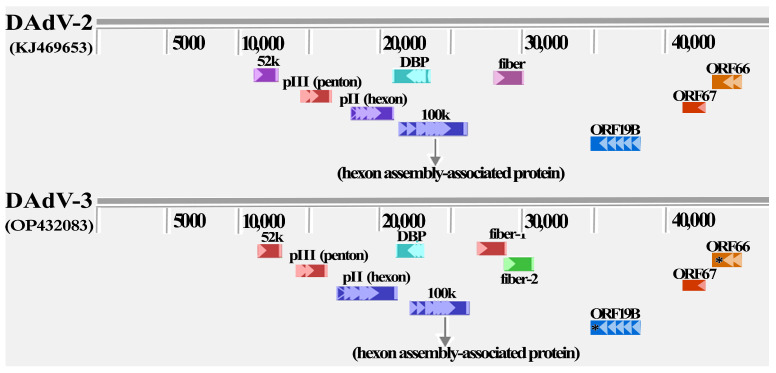
Location of major structural proteins in the DAdV-2 and 3 genomes. DAdVs contain hexon, fiber, penton, 52 K, 100 K, and DBP (DNA binding protein), but the position and sequence identity differ, especially between DAdV-2 and 3. The black asterisk marks the major regions of difference between different virulent DAdV-3 strains.

The genome sequence of DAdV-3 was obtained by sequencing. The complete amplified genome of HF-AH-2022 was submitted to GenBank under the accession number OP432083. A comparative analysis of DAdV-2 (KJ469653) and DAdV-3 (OP432083) is shown in Figure 2. From the map of the proteins encoded by the virus, the main structural proteins encoded by DAdV-2 and 3 were 52 K, penton, hexon, 100 K hexon assembly-associated protein, DBP, and fiber, among which, DAdV-3 has two fiber proteins named Fiber 1 and Fiber 2. The whole-genome sequencing analysis revealed that DAdV-3’s nucleotides were 92.3% homologous to those of DAdV-2. The amino acid sequence identity of hexon, Fiber 1, and Fiber 2 of DAdV-3 and DAdV-2 was 86.9%, 15.3%, and 28.5%, respectively.

By comparing the two strains of DAdV-3 (HF-AH-2020 and HF-AH-2022) that had different levels of virulence, both of which were isolated in this laboratory, the main difference was found in ORF19B and ORF66 [9]. Compared with HF-AH-2020, HF-AH-2022 showed a deletion of two threonines at Positions 937 and 938 in ORF19B, while HF-AH-2022 exhibited a premature termination of the translation proteins at Position 157 in ORF66. The mutant and missing amino acids are marked with a black asterisk in Figure 2. However, after SPF chickens in the experimental group were infected with DAdV-3 (HF-AH-2022) derived from Muscovy ducks, no mutation of the amino acids was observed in the major mutation regions (ORF19B, ORF66, and ORF67) of DAdV-3 (OP432083).

**Figure 3:**
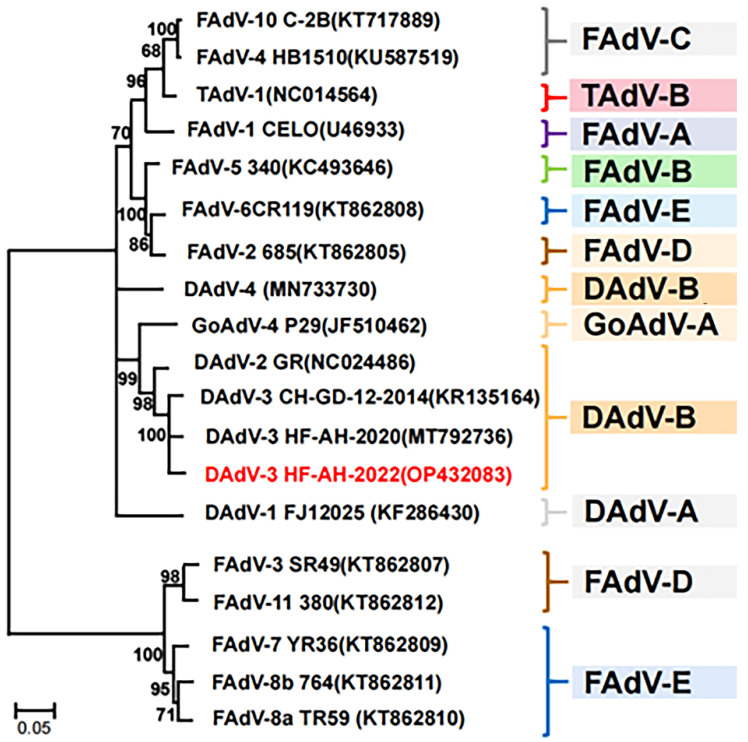
The phylogeny based on the *hexon* gene of adenoviruses, as indicated by their accession numbers. The evolutionary history was inferred using the neighbor-joining (NJ) method. For the analysis using MEGA 11, the percentages of the relevant taxon clusters in the bootstrap test (1000 replicates) are shown below the branches.

Phylogenetic analysis showed that the *hexon* gene in the amino acid sequence of HF-AH-2022 (OP432083) shared a high level of sequence identity with the previously reported HF-AH-2020 (MT792736), CH-GD-12-2014 (KR135164), and DAdV-2 (NC024486) of 100%, 100% and 86.9%, respectively, and it belonged to the same branch. However, it shared a low level of sequence identity with DAdV-1 (KF286430), FAdV-4 (KU587519), DAdV-4 (MN733730), and GoAdV-4 (JF510462) of 52.8%, 65.2%, 72.3%, and 82.4%, respectively, and belonged to a different branch, as shown in Figure 3.

### 3.3. The Level of Antibodies

**Figure 4:**
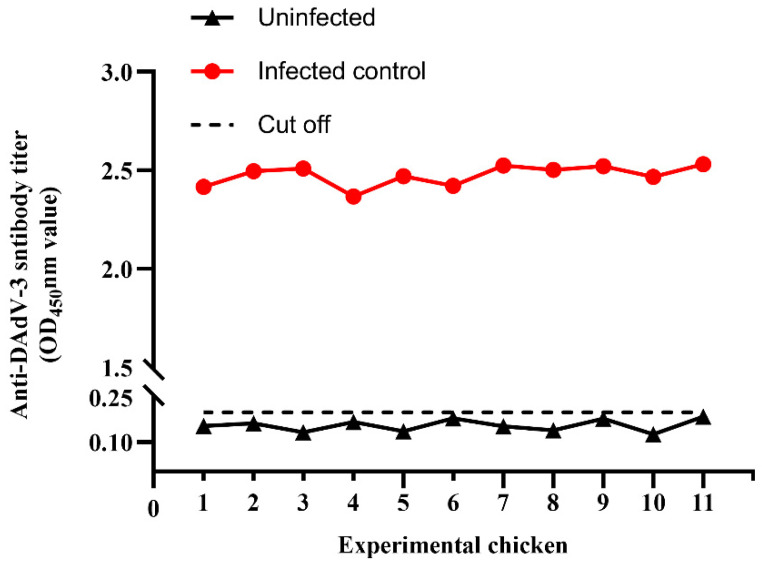
Levels of DAdV-3 antibodies in the chickens in the infected group (Group 8) and in the control group (Group 10) within the same 12-day post-infection period (dpi).

First, the positive and negative OD values were determined according to the ELISA method. The OD450 values for negative and positive values were 2.56 and 0.130, respectively, so the maximum value of P/N was 19.69. Secondly, the antibody levels of the experimental group were measured. After 12 days of inoculation, 0.5 mL of blood was collected from the surviving chickens in Group 8 (n = 11). Meanwhile, the blood of negative chickens in Group 10 (n = 11) was used as a control. When the dilution of the experimental chicken serum reached 1:20,000, the OD450 nm value of the surviving chickens was found to range from 2.41 to 2.54, while that for the control group ranged from 0.125 to 0.2, and there was no significant difference between the same groups of chickens. The positive rate for DAdV-3 antibodies in the infected group was 100%, and the level of antibodies was higher than that in the control group (Figure 4). The P/N values of these 11 chickens (Group 8) were 20.25, 20.23, 23.27, 13.28, 23, 12.7, 18.29, 23.09, 12.8, 25.5, and 12.8.

### 3.4. Lethality of Chickens, Symptoms, Macroscopic Lesions, and Histopathological Analysis

To verify the pathogenicity of the virus, the SPF chicks were infected with viruses derived from chicken embryos and duck embryos. Chickens infected via subcutaneous inoculation began to die on the third day, and all died within 5 days (Groups 1 and 2). In the case of eye–nose drop inoculation, the mortality rate was 0 in chickens that received a single inoculation (Groups 5 and 7), while the mortality rate was 26.7% in those that received multiple consecutive inoculations (Groups 6 and 8), as shown in Table 3. Meanwhile, the mortality rate of 15- and 30-day-old chicks inoculated via injection with allantoic fluid from duck embryos was 100%, as shown in Table 3 (Groups 11 and 12), whereas the control group remained healthy (Groups 3, 4, 9, 10, 13, and 14).

**Figure 5:**
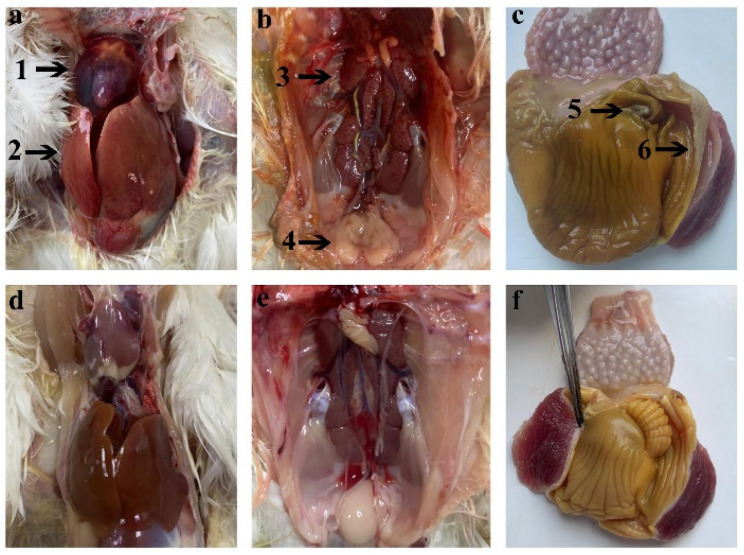
Clinical signs and gross lesions of DAdV-3 HF-AN-2022-infected chickens and uninfected controls. (**a**) Yellow jelly or watery-like exudate found in the pericardial sac (black arrow 1), Liver enlargement and bleeding (black arrow 2). (**b**) Swollen and petechial hemorrhages of the kidney (Black arrow 3) and bursa (black arrow 4). (**c**) The gizzard’s endothelium underwent focal necrosis, ulcerated (black arrow 5), fell off (black arrow 6). (**d**–**f**) The pericardia, livers, kidneys, and bursa of chickens from the control group.

In order to analyze the main lesions and pathological changes in the organs after the viral infection, necropsies were conducted on the dead chicks. Each group comprised three chicks subjected to a necropsy, and the findings are summarized in Table 3. The most prevalent symptom was hepatitis–hydropericardium syndrome (HHS). During the necropsy, the pericardium produced a light-yellow jelly or water-like transparent exudate, as shown in Figure 5a, whereas the liver, kidneys, and bursa were swollen with petechial hemorrhages; see Figure 5b. In addition, the gizzard’s endothelium fell off and ulcerated, and focal necrosis occurred, as shown in Figure 5c. No clinical symptoms or pathological lesions were observed in the control group, as shown in Figure 5d–f.

**Figure 6:**
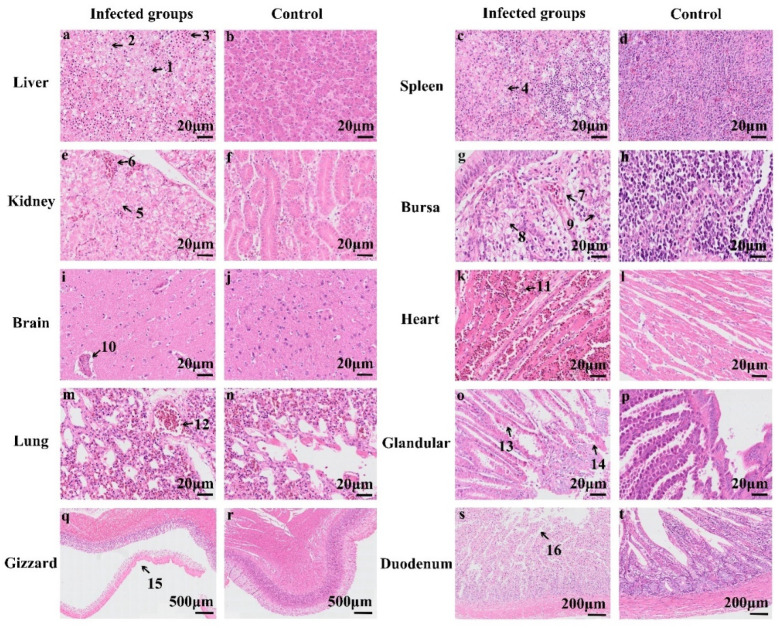
Histological changes in the organs of chickens infected with DAdV-3 (HF-AN-2022) and in the uninfected control group. In the infected group, we observed (**a**) liver cell necrosis, bleeding, and basophilic inclusion bodies. The lower left corner shows an enlarged image of the inclusion body (bar = 20 µm). (**c**) Necrosis of spleen cells, karyolysis, and pycnosis occurred in the nucleus (bar = 20 µm). (**e**) Swelling, bleeding, and necrosis of tubular epithelial cells in the kidney (bar = 20 µm). (**g**) Lymphocyte necrosis and microvascular congestion in the follicles of the bursa, with basophilic inclusion bodies (bar = 20 µm). (**i**) Cerebral congestion (bar = 20 µm). (**k**) Cardiac hemorrhage (bar = 20 µm). (**m**) Congestion in the pulmonary small vessels of the lungs (bar = 20 µm). (**o**) Necrosis and severe exfoliation of adenogastric epithelial cells (bar = 20 µm). (**q**) Dissection of the gizzard’s endothelium (bar = 500 µm). (**s**) Necrosis and exfoliation of duodenal epithelial cells of the challenge group (bar = 200 µm). (**b**,**d**,**f**,**h**,**j**,**l**,**n**,**p**,**r**,**t**) Liver, spleen, kidney, bursa, brain, heart, lung, glandular stomach, gizzard, and duodenum in the negative control group. Bar = 20–500 µm.

All dead chickens infected with HF-AN-2022 in Groups 1 and 2 exhibited severe histopathological changes in various tissues compared with the controls. Organs were harvested from three chicks from each group for the purpose of pathological observations, and the main findings are summarized in Table 3. The main histopathological changes are shown in Figure 6, which depicts changes in the liver, spleen, kidney, bursa, brain, heart, lung, glandular stomach, gizzard, lung, and duodenum. The most obvious changes were swelling, necrosis, lymphocyte infiltration, and basophilic inclusion bodies in multiple organs.

The main pathological changes in the dissected chickens were liver cell necrosis (Figure 6a, Arrow 1), basophilic inclusion bodies (Figure 6a, Arrow 2), enlarged basophilic inclusion bodies (Figure 6a, lower left corner), and liver hemorrhage (Figure 6a, Arrow 3); karyolysis and pyretosis of the spleen cells (Figure 6c, Arrow 4); necrosis (Figure 6e, Arrow 5), swelling, and bleeding (Figure 6e, Arrow 6) of the tubular epithelium of the kidney; lymphocyte necrosis (Figure 6g, Arrow 8) in the lymphoid follicles of the bursa; intravascular congestion (Figure 6g, Arrow 7) and basophilic inclusion bodies (Figure 6g, Arrow 9); cerebral congestion (Figure 6i, Arrow 10); cardiac hemorrhage (Figure 6k, Arrow 11); congestion in the pulmonary small vessels of the lungs (Figure 6m, Arrow 12); necrosis (Figure 6o, Arrow 13) and severe exfoliation (Figure 6o, Arrow 14) of the adenogastric epithelial cells; an easy-to-peel intima of the gizzard (Figure 6q, Arrow 15); and necrosis and exfoliation of epithelial cells in the duodenal challenge group (Figure 6s, arrow 16). No pathological changes were observed in the slices of the control group (Figure 6b,d,f,h,j,l,n,p,r,t).

In summary, HF-AN-2022 can cause severe histopathological changes in various tissues in chickens, indicating that the virus attacks multiple organs, leading to pathological changes.

### 3.5. Distribution of the Virus in Chicken’s Organs

**Figure 7:**
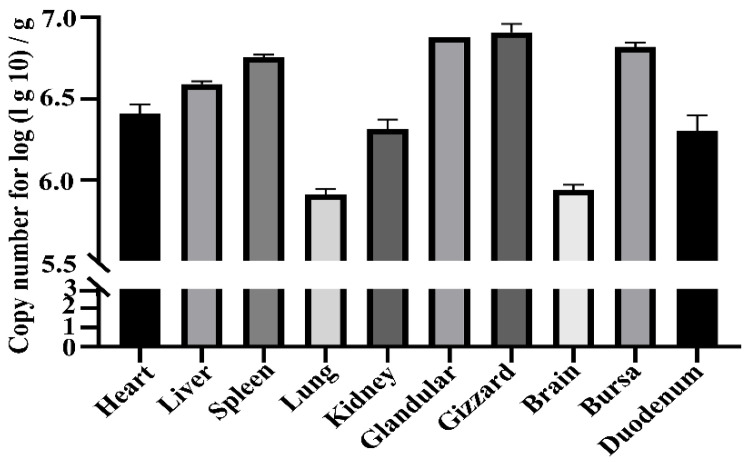
This bar graph was created after sampling the bottom 10 log (lg 10)/g tissue. Note: the results indicate the distribution of the virus in different tissues (Group 2). The viral loads in the gizzard, glandular stomach, bursa, spleen, and liver were highest at 8.1 × 10^6^, 7.6 × 10^6^, 6.6 × 10^6^, 5.7 × 10^6^, and 3.9 × 10^6^ copies/g, respectively, followed by those in the heart, kidney, and duodenum at 2.6 × 106, 2.1 × 10^6^, and 2 × 10^6^ copies/g, respectively. The lowest viral load was in the lungs at 8.2 × 10^5^ copies/g and the brain at 8.7 × 10^5^ copies/g. Data are represented as te means ± SEM (n = 3).

Three chickens in Group 2 and 10 organs were extracted from each chicken to calculate the viral load of different tissues. The plasmid pMD18-T- DAdV-3-fiber-2 was constructed with the target gene *fiber-2*, with a standard curve of y = −3.669x + 37.53 (R2 = 0.98). The dissolution curves of the standard plasmids showed high specificity and efficiency. The amplified CT values for each infected organ were incorporated into the standard curve to calculate the copy number of the virus in different organs. The viral loads in the gizzard area, glandular stomach, bursa, spleen, and liver were the highest, at 8.1 × 10^6^, 7.6 × 10^6^, 6.6 × 10^6^, 5.7 × 10^6^, and 3.9 × 10^6^ copies/g, followed by those in the heart, kidney, and duodenum at 2.6 × 10^6^, 2.1 × 10^6^, and 2 × 10^6^ copies/g, respectively. The lowest viral load was found in the lungs at 8.2 × 10^5^ copies/g and in the brain at 8.7 × 10^5^ copies/g, as shown in Figure 7.

## 4. Discussion

Adenovirus infection prevails among wild birds and poultry, with many serotypes. Both FAdV and DAdV can achieve cross-species transmission; for example, FAdV-4, -8b, and -11 can infect chickens, ducks, and geese [4,21,22,23,24,25]. Duck-derived adenoviruses have a severe impact on chickens. Studies have found that the adenoviruses DAdV-1 (=EDSV) and -3 isolated from ducks can infect poultry, causing with higher morbidity and mortality rates [3,26]. DAdV-1 does not cause typical clinical symptoms in ducks but causes egg-drop syndrome in chickens, resulting in higher mortality and reduced egg production in laying hens [26]. In this study, the virus from chicken or duck embryos was 100% lethal to chickens when delivered via subcutaneous inoculation. Although single eye–nose drop inoculation did not cause death in chickens, multiple inoculation caused a death rate of 26.7%. Mucosal immunity plays an important role in resistance to DAdV-3 infection. Mucosal immunity induced by a single small dose of eye–nose drops could protect chickens from infection, but several small doses of eye–nose drops caused some chickens to be unable to avoid infection and thus led to death. It is therefore recommended that chickens and ducks should be separated in clinical practice, and infected chickens should be promptly detected and eliminated from flocks that have already experienced an epidemic.

DAdV-3 can infect multiple organs in chickens. The main manifestation of DAdV-3 infection in chicks was HHS, and the main pathological changes were pericardial effusion and a swollen liver, kidneys, spleen and bursa, with petechial hemorrhages. The endothelium of the gizzard was easily removed, ulcerated, and exhibited focal necrosis (Figure 5). Pathological sections revealed obvious lesions in these organs, such as cell swelling, necrosis, and basophilic inclusion bodies (Figure 6). The symptoms of HHS were the same as those caused by FAdV-4 infection [27], but unlike FAdV-4 infection, DAdV-3 infection can cause the gizzard’s endothelium to fall off easily. To avoid this phenomenon, which may be caused either by FAdV-4 or a combination of FAdV-4 and DAdV-3, this study designed two primers to amplify the *fiber-2* and *hexon* genes of DAdV-3 during the identification of samples with liver disease. Meanwhile, the whole genome of the liver-extracted virus was sequenced. The results showed that only DAdV-3-specific target bands were amplified during PCR amplification, but no FAdV-4 bands were detected (Figure 1).

Cells in most other organs, such as the spleen, heart, lung, glandular stomach, and duodenum, also showed obvious lesions (Figure 6). The qPCR results showed that the virus was distributed in many organs, especially those with obvious pathological sections, such as the gizzard, glandular stomach, bursa, spleen, and liver, followed by the heart, kidney, and duodenum (Figure 7). Despite the obvious pathological changes, the viral load in the organs was not very high; it was similar to the viral load of FAdV-4 in the organs of ducks [28]. The hexon protein of the virus binds to the receptor of the host cell (CCT7) and the initiator of host-cell autophagy(BAG3), facilitating its replication within the cell [29,30]. Among these pathological changes, the thinning of the aortic vessel wall had potential significance, as it could lead to changes in the permeability of the blood vessels and allow the leakage of cytokines, resulting in pericardial effusion and death in chickens. It has also been suggested that cytokine storms are the direct cause of death from avian adenoviruses, similar to other virus-related deaths in chickens [31,32,33,34,35,36]. Multiple immunological cytokines (IL-1, TNF-a, IL-8, IL-6, IFNs, Mx, and OAS) were shown to have elevated mRNA expression in chicken tissues infected with hypervirulent FAdV-4 [18,28,37]. Ducks are more resistant to adenoviruses, possibly because they produce fewer cytokines such as IL-6 and -8, compared with chickens, and also because of duck RIG-1 [38]. Therefore, most FAdV4 strains generally infect ducks, and some strains (SD0828) can replicate in ducks and DEF cells without producing typical symptoms [28].

Amplification and comparison of the major mutation regions ORF19B, ORF66, and ORF67 in chickens and Muscovy ducks infected with the HF-AN-2022 revealed no mutation in these regions. Further comparison with the gene sequences of other published DAdV-3 strains such as HF-AH-2020 and GD-CH-2014 revealed that two threonines were missing in ORF19B of the HF-AH-2022 strain, and ORF66 also showed a premature termination of translation. A similar phenomenon was found in the GD-CH-2014 strain [39], but whether these affect virulence remains to be studied. Amino acid mutations may be a result of recombination between adenoviruses, or, alternatively, they may be caused by differences in the variety of infected hosts. In China, recombination events have been reported, and the main recombination genes are *hexon*, *fiber*, and *ORF19B* [40,41]. This change in DAdV-3 is different from that in FAdV because the main recombinant region of FAdV (D and E) are in the *hexon* and *fiber* genes, the right-terminal ORFs 19 and 25, and the ORF20/20A family [42].

In follow-up research, we found that although the virus was virulent after infecting chickens, the virulence decreased with the passage of time. The major mutation areas were amplified again, and these areas remained unchanged. This indicated that although HF-AN-2022 may lead to a decline in virulence during in vitro transmission, different virulent strains will be generated as a result, and this effect requires further study. We also discovered that FAdV-4 caused a decrease in virulence during the process of passage, and when FADV passed to the 80th generation, the virulence decreased as a result of amino acid mutations in Fiber 2 [43,44]. It is currently unclear whether this is common, and, to the best of our knowledge, this is the first report on the pathogenicity of DAdV-3 in chickens, which is of great significance for the prevention and control of DAdV-3.

## 5. Conclusions

DAdV-3 isolated from Muscovy ducks can infect chickens, causing classic hepatitis–hydropericardium syndrome (HHS) with ulceration of the glandular stomach. In our experiments, after infection, the bursa was swollen with petechial hemorrhages. DAdV-3 infection occurred in most of the chickens, and the gizzard area, glandular stomach, bursa, spleen, and liver were found to carry the highest amount of the virus. Surviving chicks exhibited extremely high levels of antibodies. Histopathological examinations showed swelling, necrosis, lymphocyte infiltration, and basophilic inclusion bodies in multiple organs. The mortality rate caused by DAdV-3 was related to the route of inoculation. The impact of the route of infection was also important for DAdV-3. The mortality rate was 100% for subcutaneous injection in the neck and 26.7% for inoculation via multiple eye–nose drops. The major variation domains (ORF19B, ORF66, and ORF67) did not change after infection with the virus in Muscovy ducks and chickens.

## Figures and Tables

**Table 2:** Experimental inoculation of the chicken virus from allantoic fluid.

Inoculated Animals	Subcutaneous Inoculation		Eye–Nose Drop Inoculation	
	Groups	Dose and Frequency	Groups	Dose, Frequency, and Days
15-day-old SPF chickens	Group 1	0.2 mL virus/time, once	Group 5	0.2 mL virus/time, once
15-day-old SPF chickens			Group 6	0.2 mL virus/time, twice a day for 3 days
30-day-old SPF chickens	Group 2	0.2 mL virus/time, once	Group 7	0.2 mL virus/time, once
30-day-old SPF chickens			Group 8	0.2 mL virus/time, twice a day for 3 days
15-day-old SPF chickens	Group 3	0.2 mL PBS, once	Group 9	0.2 mL PBS/time, twice a day for 3 days
30-day-old SPF chickens	Group 4	0.2 mL PBS, once	Group 10	0.2 mL PBS/time, twice a day, for 3 days
15-day-old SPF chickens	Group 11	0.2 mL virus, once		
30-day-old SPF chickens	Group 12	0.3 mL virus, once		
15-day-old SPF chickens	Group 13	0.2 mL, PBS, once		
30-day-old SPF chickens	Group 14	0.3 mL, PBS, once		

**Table 3:** Intensity and distribution of gross lesions and histopathology.

	Tissue	15 d (Group 1)	30 d (Group 1)	Control (15 d) (Group 3)	Control (30 d) (Group 4)
Chicken No.		1 2 3	4 5 6	7 8 9	10 11 12
Gross lesions	Heart	+++, ++, ++	++, ++, ++	–, –, –	–, –, –
	Liver	+++, +++, +++	+++, +++, +++	–, –, –	–, –, –
	Spleen	+, +, +	+, +, +	–, –, –	–, –, –
	Lung	–, –, –	–, –, –	–, –, –	–, –, –
	Kidney	+++, +++, +++	+++, +++, +++	–, –, –	–, –, –
	Glandular	–, –, –	–, –, –	–, –, –	–, –, –
	Gizzard	++, ++, ++	++, ++, ++	–, –, –	–, –, –
	Brain	–, –, –	–, –, –	–, –, –	–, –, –
	Bursa	+++, +++, +++	+++, +++, +++	–, –, –	–, –, –
	Duodenum	–, –, –	–, –, –	–, –, –	–, –, –
Histopathology	Heart	+++, +++, +++	+++, ++, +++	–, –, –	–, –, –
	Liver	+++, +++, +++	+++, +++, +++	–, –, –	–, –, –
	Spleen	++, ++, ++	++, ++, ++	–, –, –	–, –, –
	Lung	+, ++, +	+, +, +	–, –, –	–, –, –
	Kidney	+++, +++, +++	+++, +++, +++	–, –, –	–, –, –
	Glandular	+, ++, +	+, +, +	–, –, –	–, –, –
	Gizzard	+++, +++, +++	+++, +++, +++	–, –, –	–, –, –
	Brain	–, –,–	–, –,–	–, –, –	–, –, –
	Bursa	+++, +++, +++	+++, +++, +++	–, –, –	–, –, –
	Duodenum	+++, +++, +++,	+++, +++, +++	–, –, –	–, –, –

Chicken Nos. 1 to 6 represent the number of infected chickens; chicken Nos. 7 to 12 represent the number of control chickens. Gross lesions: –, absent; +, present. Scoring of histopathology: –, normal + congestion, hemorrhage, and mononuclear cell infiltration; +, mild <20%; ++, moderate <50%; +++, severe >50% necrosis.

## Data Availability

All materials, data, and associated protocols will promptly be made available to readers, without undue qualification, in line with the material transfer agreements.
